# An injectable 2.5% cross-linked polyacrylamide hydrogel (2.5 iPAAG) demonstrates no neurotoxicity in human induced pluripotent stem cells-derived iCell^®^ GlutaNeurons

**DOI:** 10.3389/ftox.2025.1585430

**Published:** 2025-06-23

**Authors:** Peter S. Walmod, Philip Kusk, Nina Jøhnk, Ieva Ankorina-Stark, Anthony Essex

**Affiliations:** ^1^ Contura International A/S, Soeborg, Denmark; ^2^ Phenovista Biosciences, San Diego, CA, United States

**Keywords:** acrylamide, Arthramid^®^, Arthrosamid^®^, Bulkamid^®^, Mictamid^®^, neurotoxicity, polyacrylamide

## Abstract

**Introduction:**

Arthrosamid^®^, Arthramid^®^, Bulkamid^®^, and Mictamid^®^ are products for the management of osteoarthritis and female urinary incontinence. All four products include the same injectable hydrogel consisting of 2.5% crosslinked polyacrylamide termed 2.5 iPAAG that is polymerized from the neurotoxic compound acrylamide.

**Methods:**

To investigate whether 2.5 iPAAG demonstrates any neurotoxic effects *in vitro*, human iCell^®^ Glutaneurons were exposed to concentrations of up to 20% (w/w) 2.5 iPAAG for up to 96 h. Cells were stained and recorded by fluorescence microscopy for the subsequent estimation of cell survival, cell death, apoptosis, and the formation and maintenance of the neurite network.

**Results:**

The negative control, Fish Gelatin, did not affect cell survival, cell death, or apoptosis, and had no or minor effects on the neurite network area. The positive controls acrylamide and A23187 caused a pronounced time- and dose-dependent decrease in cell survival, an increase in cell death, but not apoptotic cell death, and a strong decrease in neurite network area, whereas another positive control, Tunicamycin, caused a time- and dose-dependent increase in cell death and apoptosis but only a minor decrease in the average neurite network area. At the tested concentrations and timepoints, the 2.5 iPAAG had no statistically significant effects on cell survival, non-apoptotic and apoptotic cell death, or the neurite network area.

**Discussion:**

These results support the interpretation that 2.5 iPAAG demonstrates no apparent *in vitro* neurotoxic or cytotoxic effects in human iCell^®^ Glutaneurons when included in cell cultures at concentrations of up to 20% (v/v) for up to 96 h.

## 1 Introduction

Arthrosamid^®^ is a human medical device intended for symptomatic treatment of adult patients with knee osteoarthritis (Bliddal et al., 2024). Arthramid^®^ is the veterinary equivalent to Arthrosamid^®^ intended for the management of non-infectious causes of joint disease in horses and dogs, including early and late stages of osteoarthritis (de Clifford et al., 2019; de Clifford et al., 2021).

Bulkamid^®^ is a human medical device for the management of female urinary incontinence where the stress component is significant (Osse et al., 2024), and Mictamid^®^ is the veterinary equivalent intended for the treatment of urinary incontinence in female dogs, including Urethral Sphincter Mechanism Incompetence (USMI).

Arthrosamid^®^ and Arthramid^®^ are injected intra-articularly, where the iPAAG becomes incorporated into the synovial lining (Christensen et al., 2016). Bulkamid^®^ and Mictamid^®^ are injected into the submucosal tissue of the proximal half of the urethra, where the products improve urethral coaptation through bulking (Hoe et al., 2021).

Despite different modes of action, Arthrosamid^®^/Arthramid^®^ and Bulkamid^®^/Mictamid^®^ contain the same active substance, an injectable hydrogel termed 2.5 iPAAG, consisting of 2.5% crosslinked polyacrylamide and 97.5% water for injection. During production of the 2.5 iPAAG monomeric acrylamide (AM) and monomeric N,N′-methylenebisacrylamide (MBAM) polymerize to form the cross-linked hydrogel.

Treatment with Arthrosamid^®^ and Arthramid^®^ are characterized by reduced pain, documented as a general reduction in the WOMAC pain score and the AAEP lameness score in humans and horses, respectively (Bliddal et al., 2024; de Clifford et al., 2019; de Clifford et al., 2021). However, despite a very low residual concentration of AM in 2.5 iPAAG regulatory authorities have occasionally questioned whether the beneficial effects of Arthrosamid^®^ and Arthramid^®^ are due to a neurotoxic effect of residual AM leading to a reduction in pain through a degeneration of sensory neurons. Therefore, it is important to determine whether the 2.5 iPAAG has any neurotoxic effects *in vitro*.

*In vitro* neuronal cultures have traditionally utilized established immortalized cell lines or primary cultures derived from rodents. However, in recent years there has been a progress in the design of neuronal model systems that improves the translational value of the obtained data (Nikolakopoulou et al., 2020). One option is the use of human induced Pluripotent Stem Cells (hiPSC)-derived neurons as a model system for neuronal processes mimicking both early and late stages of human CNS development (Cassiano, 2017).

Human iCell^®^ GlutaNeurons are commercially available neurons derived from hiPSCs. The cells are a mixture of different post-mitotic glutamatergic neuronal subtypes, predominantly cortical neurons, and are recommended for, *e.g.*, toxicity testing and drug screening in 2- and 3-dimensional culture models (Cellular_Dynamics_International, 2017; Yafei, 2018). The cells have been used to determine a range of endpoints including neuronal cell viability, apoptosis, neurite outgrowth, total neurite area, neuronal excitation, and Ca^2+^ oscillations (Cheng et al., 2024; Díaz et al., 2024; Imredy et al., 2023; Overland et al., 2017; Yafei, 2018), and they have been used in studies of, *e.g.*, neuronal excitotoxicity, including amyotrophic lateral sclerosis-linked excitotoxicity (Cheng et al., 2024; Overland et al., 2017), the effects of Aβ42 oligomers in the pathogenesis of Alzheimer’s disease (Díaz et al., 2024), neuropharmacological safety screening (Imredy et al., 2023), and the neurotoxic effects of polyfluoroalkyl substances (PFAS) on spontaneous neuronal networks activity (Tukker Anke et al., 2020).

The aim of this study was to investigate whether 2.5 iPAAG demonstrates any neurotoxic effects *in vitro*, by exposing human iCell^®^ Glutaneurons to different concentrations of the hydrogel for up to 96 h.

## 2 Materials and methods

All experiments were performed by PhenoVista Biosciences (San Diego, CA, United States).

### 2.1 Cell culture

On Day 1, iCell^®^ GlutaNeurons (FUJIFILM Cellular Dynamics, Inc. (FCDI)). were seeded in poly-L-Ornithine (PLO) and Matrigel-coated 384-well SCREENSTAR Microplates (Greiner Bio-One International GmbH) (23 × 10^3^ cells/well, 100 µL/well). Wells were coated (0.01% PLO, 15 µL/well, 1 h, R.T.). Wells were washed 3× with UltraPure water and left in a biosafety cabinet overnight to dry. Plates were wrapped in Parafilm and stored at 4°C until Matrigel coating. For the Matrigel coating, Matrigel was diluted to 0.028 mg/mL in BrainPhys Complete Media. Plates were incubated with 5 µL/well (1 h, 37°C). Neurons were then seeded. Cells were cultured in BrainPhys™ Neuronal Medium (STEMCELL™ Technologies), a serum-free basal medium based on the formulation provided by Bardy et al. (Bardy et al., 2015). The cell cultures were kept in an incubator (37°C, 5% CO_2_). On Day 2 and Day 4 the medium was refreshed by a full- and a half-volume media exchange, respectively. On Day 6, the cultures were exposed to various treatments by a full-volume medium exchange to BrainPhys™ Neuronal Medium containing the various treatments. Subsequently, the cultures were grown for 24, 48, or 96 h. Before fixation, cells were stained with DRAQ7™ (Abcam Limited) per manufacturer’s protocols, fixed by treating with 4% PFA (20 min, R.T.), and permeabilized by washing 3× with 0.03% Triton X-100. Cells were blocked with goat serum and stained with primary antibodies (rabbit anti-Cleaved Caspase-3, Cell Signaling Technology^®^; chicken anti-β3-Tubulin, Abcam Limited; 4°C, O.N.). The next day, the cells were rinsed and stained with secondary antibodies (Alexa Fluor 488 goat-anti-rabbit; AlexaFluor 568 goat-anti-chicken), and Hoechst 33342 (ThermoFisher Scientific).

### 2.2 Test compounds

In the study, 3 separate batches of 2.5 iPAAG were tested (Bulkamid^®^ lot 21F0302, 22F0903, and 22M1400; Contura International A/S, Denmark). The 2.5 iPAAG was tested in 2 concentrations: 10% (v/v) and 20% (v/v). Monomeric acrylamide (AM, CAS 79-06-1; Merck) was tested in 2 concentrations: 10% (v/v) and 20% (v/v). A23187 (CAS 52665-69-7; Cayman Chemical) was tested in 6 concentrations: 3.125, 6.25, 12.5, 25, 50, and 100 µM. Fish Gelatin (CAS 9000-70-8; Merck) was tested in 2 concentrations: 10% (v/v) and 20% (v/v). Tunicamycin (CAS 11089-65-9; Cayman Chemical) was tested in 6 concentrations: 3.125, 6.25, 12.5, 25, 50, and 100 µM.

Each treatment condition was tested in four wells/plate, and the data from each well are the sum of data from 9 images derived from separate areas of the well.

### 2.3 Microscopy

Images were acquired using a ThermoFisher CellInsight CX7 LZR HCS platform equipped with a laser light engine and Photometrics X1 CCD camera. Images were acquired in widefield mode, 2 × 2 binning (1104 pixels × 1104 pixels), laser autofocus with autofocus interval = 1 and camera gain = 2 for all channels and a ×10 objective (NA 0.4), 9 fields/well. Exposure settings were: Ch1 (Nuclei): 40 msec, ch2 (TUJ1): 150 msec, ch3 (c.caspase3): 300 msec, ch4 (DRAQ7™): 1000 msec, +3 offset.

### 2.4 Statistics

All treatments were compared to untreated control (BrainPhys™ Neuronal Medium) for the same timepoint by one-way ANOVA. If the ANOVA was significant (p < 0.05) the individual treatments were compared to untreated control for the same timepoint by Dunnett’s multiple comparison test. Statistics and bar diagrams were prepared using GraphPad Prism10 (RRID:SCR_002798). Statistics were performed on non-normalized values, but the data are presented as Mean and Standard Error of the Mean (SEM) of values normalized to the untreated control for the same timepoint.

## 3 Results

To analyze the effect of the polyacrylamide hydrogel test item, 2.5 iPAAG on neuronal cultures *in vitro* iCell^®^ GlutaNeurons were incubated in the presence of the hydrogel. As a control, cells were maintained in BrainPhys Complete Media in the absence of test compounds. As negative control for the 2.5 iPAAG, cells were incubated in the presence of Fish Gelatin, a material with a viscosity comparable to that of the 2.5 iPAAG. As a positive control cells were incubated in the presence of AM, a well-known neurotoxin (Erkekoglu and Baydar, 2014) and one of the raw materials utilized for the production of 2.5 iPAAG. As additional positive controls, an inhibitor of N-linked glycosylation, Tunicamycin and the divalent cation ionophore A23187 were selected.

**FIGURE 1:**
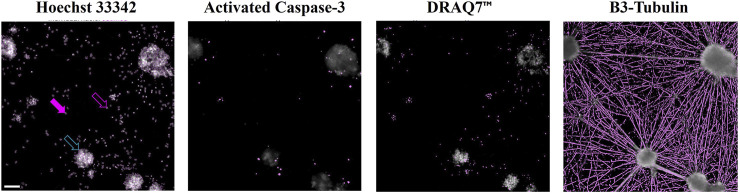
Computerized detection of signals in micrographs Cells were fixed and stained with Hoechst 33342 (top), antibodies for detection of activated Caspase-3 (second from top), DRAQ7™ (third from top), and antibodies for detection of β-Tubulin 3 (bottom row). The images show a representative region of a cell culture after identification of signal above background. Filled purple arrow: Nucleus of a live cell (large nuclear area, moderate staining signal). Open purple arrow: Nucleus of a dead cell (small nuclear area, strong staining signal). Open turquoise arrow: Cluster of nuclei of live and dead cells (large area, highest staining signal). Scale bar (top left micrograph) = 100 µm. The presented cells were grown under control conditions (media alone).

The cultured cells were stained with the DNA-binding fluorophores Hoechst 33342 and DRAQ7™, and antibodies for identification of cleaved Caspase-3 and β3-Tubulin. The individual stains are shown in representative micrographs in Figure 1 that present cells grown under control conditions. The presented culture area shows several neuronal aggregates with individual neurons interspersed. Hoechst 33342 is cell-permeable and visualizes the nuclei of both live and dead cells (Figure 1, top). The staining is used for the estimation of the total number of cells in the respective recorded culture areas. The DRAQ7™ dye is membrane impermeable. It enters only cells with compromised membranes, and the staining is used to estimate the number of dead/dying cells (Figure 1, third from top). Cleaved Caspase-3 (Figure 1, second from top) is an activated enzyme involved in apoptosis, and the staining of cleaved Caspase-3 is used to estimate the fraction of cells undergoing apoptosis. The cytoskeletal protein β3-Tubulin (Figure 1, bottom row) is neuro-specific, and the staining of β3-Tubulin is used for visualization of neurons including their neurites, and for the quantification of the neurite network generated by the cultured cells.

Quantification of the respective stains was performed in a stepwise manner where subtraction of background staining was followed by identification and quantification of signals above the respective background levels.

### 3.1 Test item comparison

In the study, 3 different batches of 2.5 iPAAG were tested. To determine whether the batches affected the cells differently, the data for all batches for each timepoint and each concentration were compared by one-way ANOVA. Out of the 24 individual analyses (2 doses × 3 timepoints × 4 stains) only one individual analysis demonstrated a statistically significant difference (DRAQ7™-staining, 10% 2.5 iPAAG, 24 h; p < 0.002431). It was therefore decided to pool the data for the 3 batches. Consequently, datapoints for 2.5 iPAAG include 12 separate measurements (4 repetitions from 3 batches) whereas all datapoints for the analysis of other treatments include 4 separate measurements.

### 3.2 Effects on the number of cells

Being terminally differentiated from hiPSCs, iCell^®^ GlutaNeurons do not proliferate. Therefore, any treatment affecting the survival of the cells will be reflected in the number of cells per well.

**FIGURE 2:**
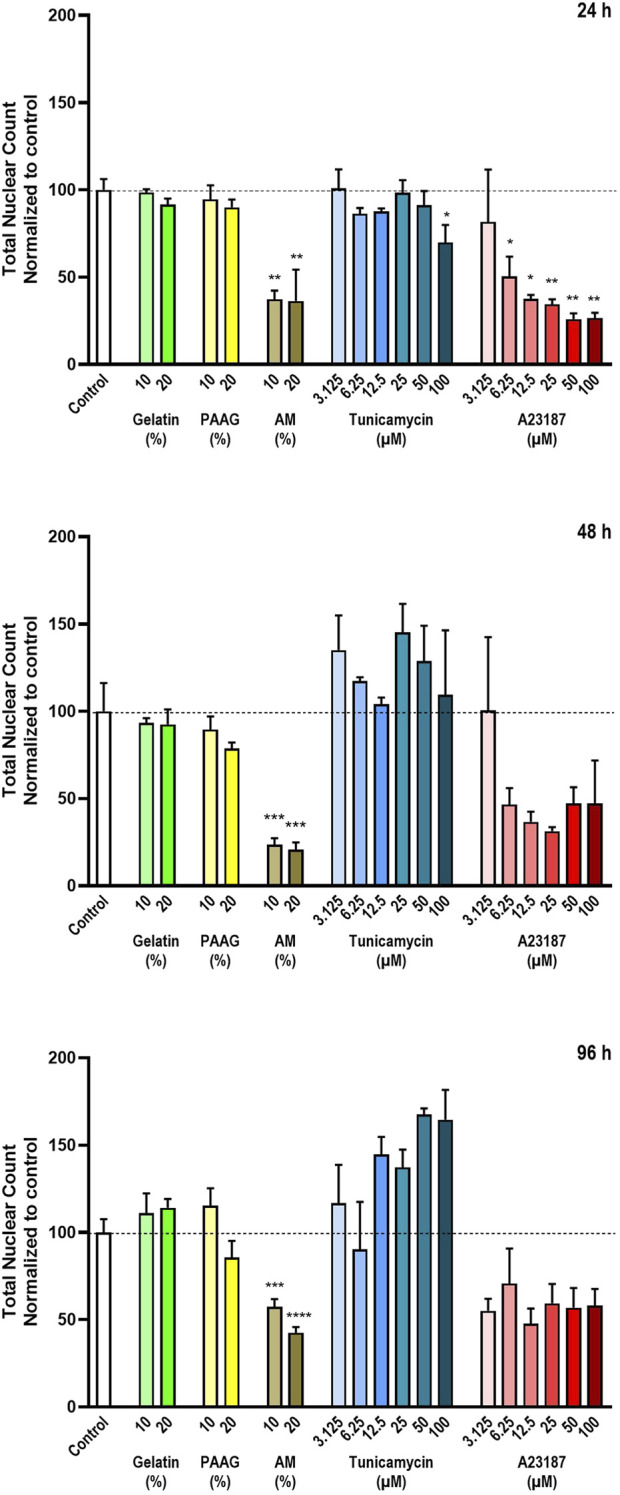
Total number of cells The total number of cells in each well was estimated by staining with the DNA-binding compound Hoechst 33342. The data are expressed as the total nuclear count per well normalized to the values in the untreated controls. Statistics were performed on non-normalized data. For each timepoint the results of the respective treatments were compared to the results from the untreated control using One-way ANOVA followed by Dunnett’s multiple comparison test. *, p < 0.01; **, p < 0.01; ***, p < 0.001; ****, p < 0.0001.

To be able to identify and quantify the total number of cells per well the cells were stained with Hoechst 33342. The quantitative results of the Hoechst 33342-stainings are presented in Figure 2, which shows the total nuclear count relative to untreated control after 24 h, 48 h, and 96 h (Figure 2, top, middle, bottom graph, respectively). Fish Gelatin had no effect at any tested timepoint or concentration (Figure 2, green bars), whereas exposure to AM and A23187 caused time- and dose-dependent decreases in the number of cells (Figure 2, brown and red bars). Tunicamycin also caused a dose-dependent decrease in the number of cells after 24 h exposure, but at later timepoints it demonstrated a tendency to cause an increase in the number of cells (Figure 2, blue bars).

The 2.5 iPAAG had no effect on the number of cells at any tested timepoint or concentration (Figure 2, yellow bars).

Cells in the process of dying will either detach from the substrate and thereby be removed from the cell culture during the staining procedure or disintegrated to a degree where detection by staining is no longer possible. In both cases, this will be detected as a decrease in the number of Hoechst-stained nuclei. However, at certain stages of, e.g., apoptosis, the dying cells may develop fragmented nuclei, and this can potentially lead to an overestimation of the number of Hoechst-stained objects and an apparent increase in the number of Hoechst-stained nuclei.

**FIGURE 3:**
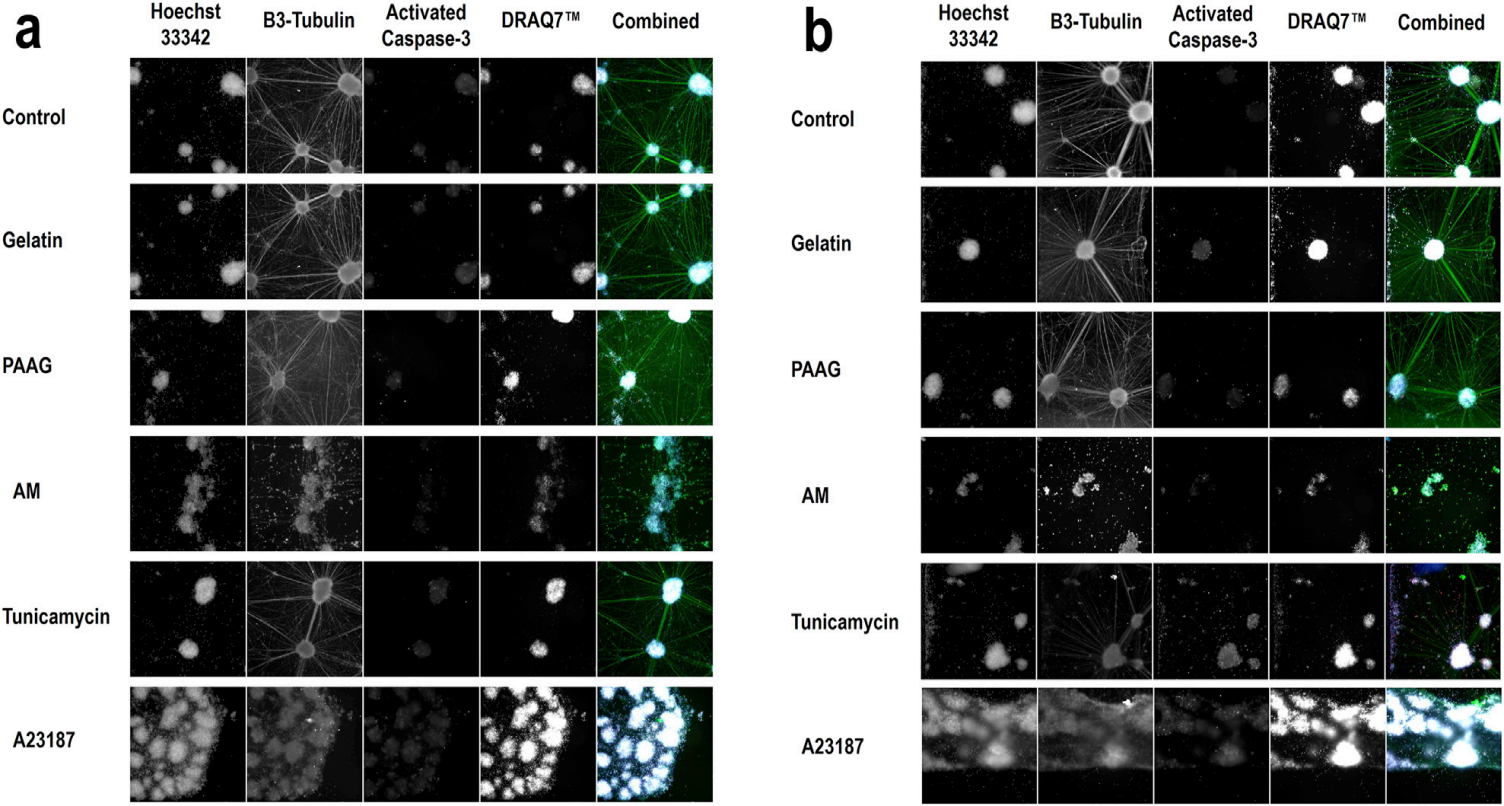
Qualitative data of selected treatments Representative images from selected concentrations of all test conditions: Control (BrainPhys™ Neuronal Medium), Gelatin (20%), PAAG (20%), AM (20%), Tunicamycin (100 μM), and A23187 (100 μM). Data from all four stains are presented as images from the individual channels (columns 1-4; Hoechst 33342, β3-Tubulin, Activated Caspase-3, DRAQ7™) and as data from all channels combined (column 5, Combined). Images from 2 timepoints are presented; 24 h incubation **(a)** and 96 h incubation **(b)**. The images in the first four columns in a and b are in greyscale. The colors of the respective stains in the combined images (column 5) are as follows: Hoechst 33342 (360 nm channel, blue), β3-Tubulin (488 nm channel, green), Activated Caspase-3 (568 nm channel, red), and DRAQ7™ (647 nm channel, white).

The apparent increase in cell numbers observed for Tunicamycin (Figure 2, 48 h and 96 h, blue bars), which cannot reflect an actual increase in the number of nuclei, since the cells do not proliferate, and the reduced response to A23187 at later timepoints (Figure 2, 48 h and 96 h, red bars) is probably an artefact caused by fragmented nuclei of apoptotic cells as described above. In Figure 3 (column 1) the Hoechst 33342-staining at selected timepoints (24 h, Figure 2, and 96 h; Figure 3b) and test conditions are presented. Treatment with high concentrations of AM, Tunicamycin, and A23187 seems to cause in increase in diffuse Hoechst 33342 stain; an effect that is even more pronounces after 96 h incubation (Figure 3b) than after 24 h incubation (Figure 3a). This effect on the Hoechst stain supports the interpretation that there is a disintegration of the nuclei under these test conditions. Moreover, cultures grown under control conditions, or in the presence of Gelatin or 2.5 iPAAG demonstrate the formation of cellular aggregates interconnected with fasciculated neurites (Figures 3a,b, last column, row 1-3). This general appearance of the cultures changes dramatically in the presence of AM and A23187, which demonstrate a complete absence of fasciculated neurites (Figures 3a,b, last column, row four and, 6). The response to Tunicamycin is less pronounced (Figures 3a,b, last column, row 5).

In summary, the Fish Gelatin and 2.5 iPAAG had no statistically significant effects on the number of cells at concentrations up to 20% (w/w) for up to 96 h of exposure, whereas AM, A23187, and to some extent Tunicamycin cause statistically significant time- and dose-dependent decreases in the number of cells.

### 3.3 Effects on cell death

DRAQ7™ stains dead and dying cells and is therefore in this study used to estimate the effects of the various treatments on cell death. However, dead and dying cells that are not stained by Hoechst because they have disintegrated or been removed from the cell culture during the staining process (as described above) will not be stained with the DRAQ7™ dye. This leads to a potential underestimation of the fraction of dead cells when the estimate is based on the quantification of the total DRAQ7™-stained area per well alone.

**FIGURE 4:**
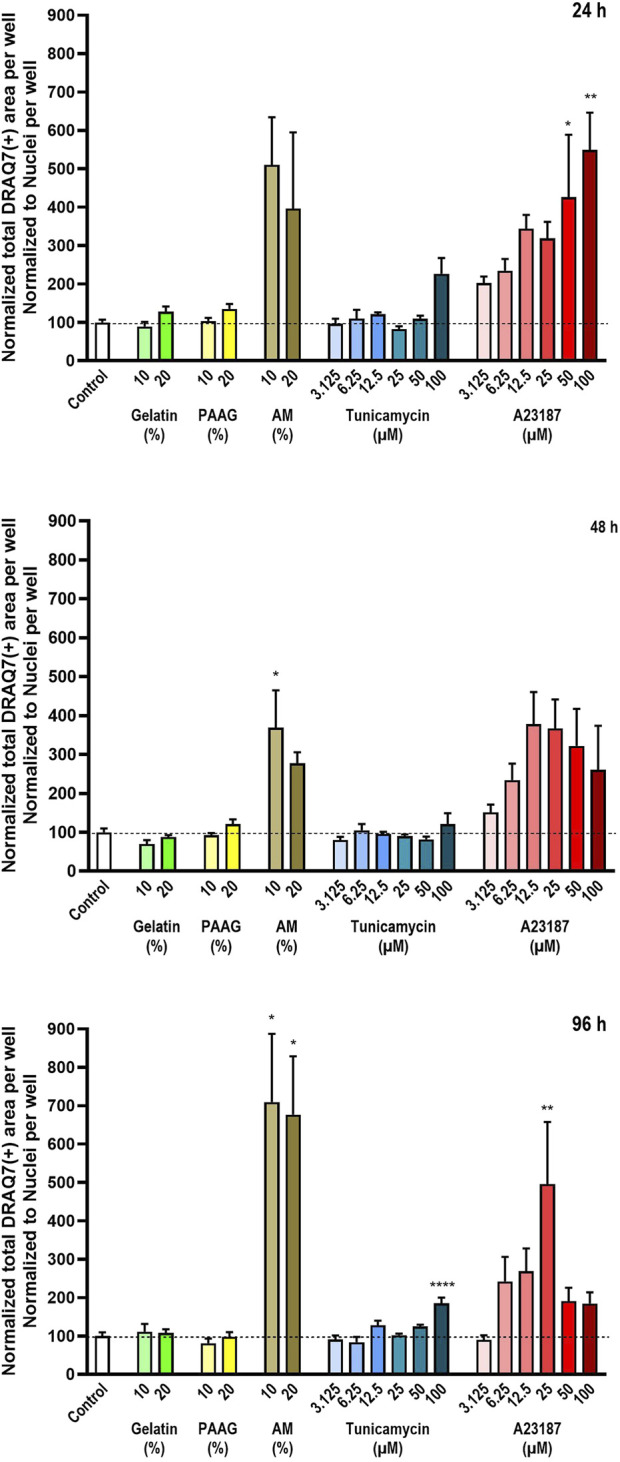
Number of dead cells relative to the total number of cells. The number of dead cells in each well was estimated by staining with the DNA-binding compound DRAQ7™. The data are expressed as the total DRAQ7™-stained area per well relative to the total nuclear count per well (Figure 2) normalized to the values in the untreated controls. Statistics were performed on non-normalized data. For each timepoint the results of the respective treatments were compared to the results from the untreated control using One-way ANOVA followed by Dunnett’s multiple comparison test. *, p < 0.01; **, p < 0.01; ****, p < 0.0001.

To compensate for the loss of dead cells from the cultures, the total DRAQ7™-stained area per well were normalized to the average number of cells per well (as presented in Figure 2). These data are presented in Figure 4. The quantified DRAQ7™ stains is therefore an estimate of the fraction of dead/dying cells still left in the cultures.

Consistent with the data presented in Figure 2 Fish Gelatin had no effect at any timepoint or concentration on the average DRAQ7™ staining per cell (Figure 4, green bars) whereas exposure to AM, A23187, and Tunicamycin caused time- and dose-dependent increases in the average DRAQ7™ staining per cell (Figure 4, brown, blue, and red bars). At high concentrations of A23187, after 96 h of incubation, there is an apparent reduction in the fraction of DRAQ7™-positive cells. This probably reflects the fact that most cells in these cultures are already dead.

The 2.5 iPAAG had no effect on the amount of DRAQ7™-staining per cell at any tested timepoint or concentration (Figure 4, yellow bars).

As shown in Figures 3a,b, column 4) all cultures contain a fraction of dead/dying cells, as demonstrated by the DRAQ7™ stain. In the presented images, this fraction is especially increased, relative to the fraction in control cultures, in the presence of A2317. In summary, exposure to Fish Gelatin and 2.5 iPAAG had no statistically significant effects on the fraction of DRAQ7™-stained cells at concentrations up to 20% (w/w) for up to 96 h of exposure, whereas all 3 positive controls caused time- and dose-dependent increases in the fraction of DRAQ7™-stained cells. This indicates that incubation in the presence of AM, Tunicamycin, and A23187, but not Fish Gelatin or 2.5 iPAAG, leads to an increase in the fraction of dead/dying cells.

### 3.4 Effects on apoptosis

Caspase-3 is a protease involved in activation of apoptosis. To become active, the Caspase-3 proenzyme needs to be cleaved by other proteases (D’amelio et al., 2010). Therefore, cleaved Caspase-3 serves as a marker for apoptosis.

**FIGURE 5:**
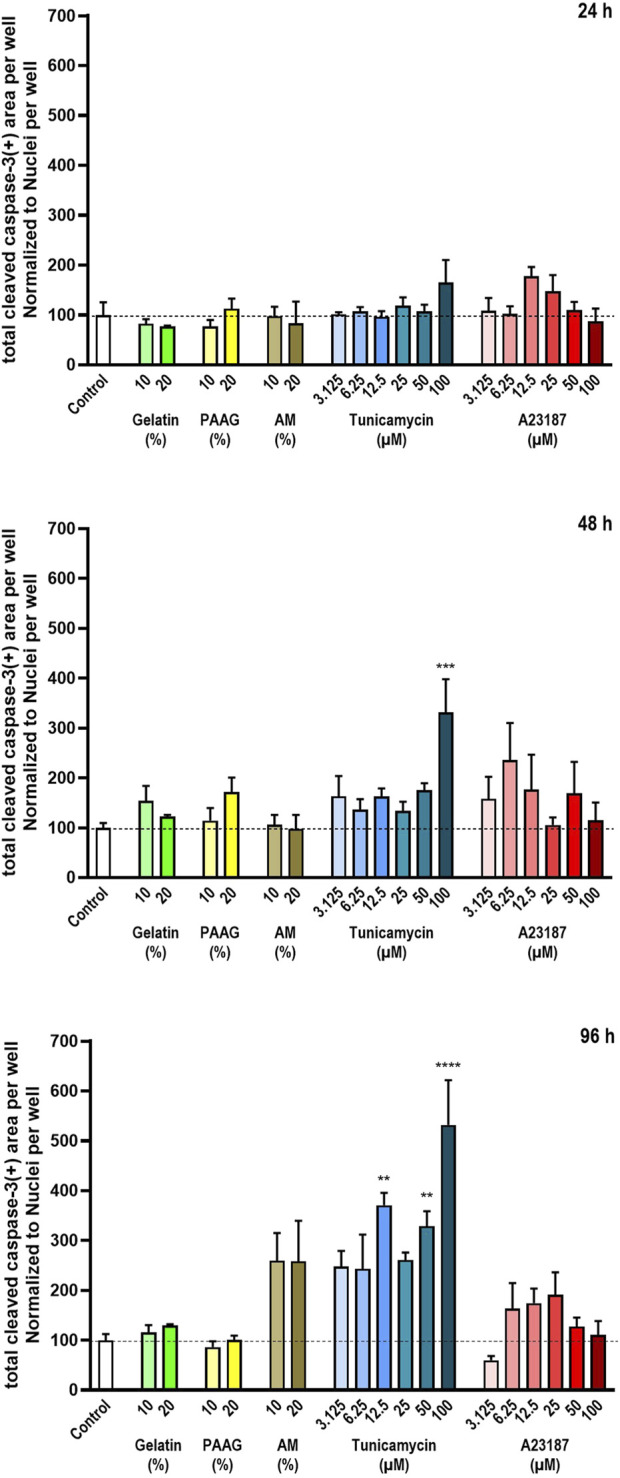
Cleaved Caspase-3 relative to the total number of cells. The degree of cleaved Caspase-3 was estimated from staining with antibodies against cleaved Caspase-3. The data are expressed as the total cleaved Caspase-3-positive area per well relative to the total nuclear count per well (Figure 2) normalized to the values in the untreated controls. Statistics were performed on non-normalized data. For each timepoint the results of the respective treatments were compared to the results from the untreated control using One-way ANOVA followed by Dunnett’s multiple comparison test. **, p < 0.01; ***, p < 0.001; ****, p < 0.0001.

Like what was done with the DRAQ7™-staining data, to compensate for the loss of dead cell from the cultures, the total cleaved Caspase-3-staining area per well was normalized to the average number of cells per well (Figure 2). The quantified Activated Caspase-3-stains is therefore an estimate of the fraction of cells undergoing apoptosis still left in the cultures. These data are presented in Figure 5.

Fish Gelatin had no effect at any timepoint or concentration on the fraction of Activated Caspase-3 in cells remaining in the cultures (Figure 5, green bars). AM and A23187 also did not cause any significant effects on the amount of Activated Caspase-3 per cell although both compounds demonstrate a tendency towards time- and dose-dependent stimulations of Caspase-3 activity (Figure 5, brown and red bars). Tunicamycin caused a pronounced time- and dose-dependent increase in the fraction of Activated Caspase-3 (Figure 5, blue bars).

The 2.5 iPAAG had no statistically significant effect on the fraction of Activated Caspase-3 in the cultures at any tested timepoint or concentration (Figure 5, yellow bars).

The time-dependent increase in the fraction of Activated Caspase-3-stained cells in cultures incubated in the presence of high concentrations of Tunicamycin is clearly noticeable in Figure 3. After 24 h incubation there is no apparent increase in the Activated Caspase-3-stain (Figure 3a, row 5, column 3), but after 96 h incubation, Activated Caspase-3-stained cells is distributed throughout the culture (Figure 3b, row 5, column 3).

In summary, at the tested concentrations and timepoints, Fish Gelatin, 2.5 iPAAG, AM, and A23187 had no statistically significant effects on the activation of apoptosis through Caspase-3 as determined by the fraction of cleaved Caspase-3-stained cells, although both AM and A23197 demonstrated a tendency towards stimulation of Caspase-3 activity. Tunicamycin caused statistically significant time- and dose-dependent increases in the fraction of Caspase-3-stained cells.

For Gelatin and the 2.5 iPAAG, where no time- or dose-dependent decrease in cell numbers have been demonstrated (Figure 2) the interpretation of the data supports the conclusion that Gelatin and 2.5 iPAAG do not induce Caspase-3-mediated apoptosis at the tested concentrations and timepoints. For AM and A23187 where time- and dose-dependent decreases in cell numbers have been demonstrated (Figure 2) the effects of these compounds on Caspase-3-mediated apoptosis as presented in Figure 5, is likely to be an underestimate, depending on whether the decrease in cell numbers presented in Figure 2 is caused by Caspase-3-mediate apoptosis or not.

### 3.5 Effects on neurite network formation

The iCell^®^ GlutaNeurons are grown for 6 days before being exposed to the test item and other treatments. During this period many of the neurons migrate to form neuronal smaller or larger aggregates, which are interconnected with fasciculated neurites. Between the neuronal aggregates individual neurons are interspersed, and together the various neurons generate a network of branching neurites (see Figure 1, bottom row images).

To test the effects of the test items on the formation and maintenance of the neurite network, the cells were stained with antibodies against the neuro-specific protein β3-Tubulin (Yuan et al., 2024) for visualization of the neurites and cell bodies.

**FIGURE 6:**
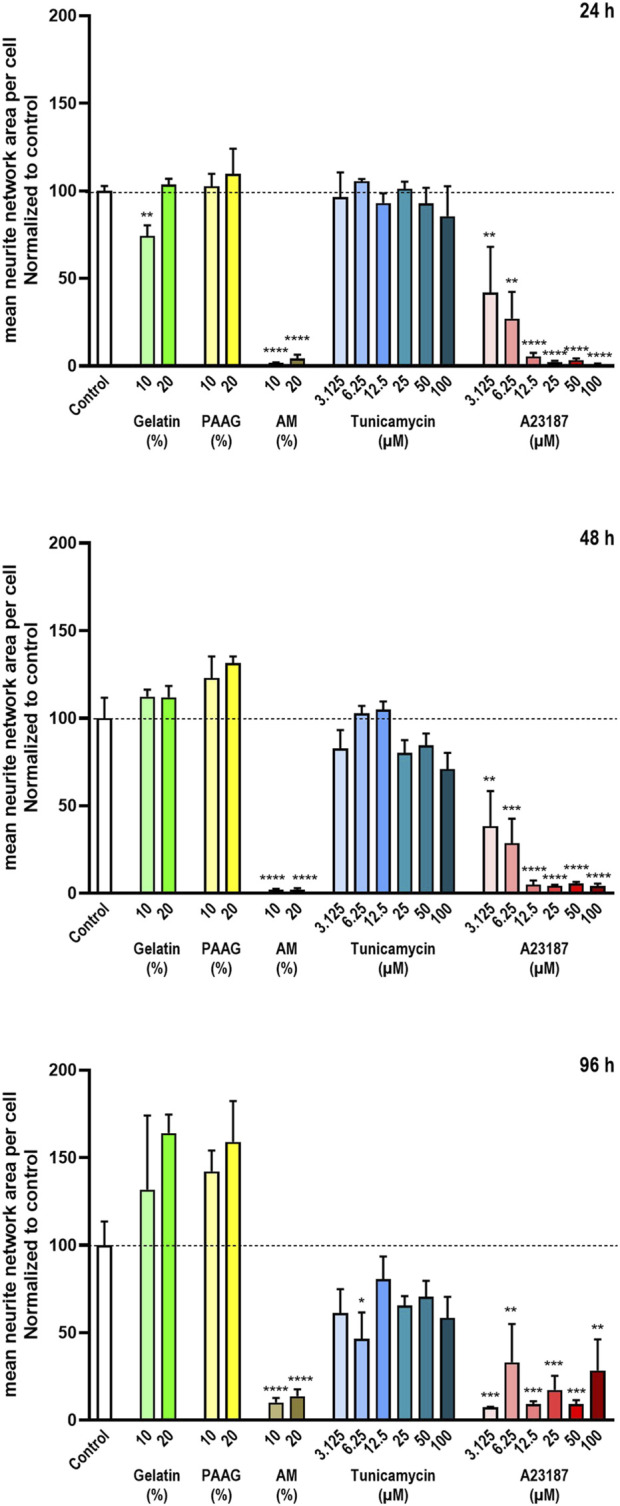
Neurite network area The neurite network area was estimated from staining with antibodies against β3-Tubulin. The data are expressed as the mean neurite network area per cell normalized to the values in the untreated controls. Statistics were performed on non-normalized data. For each timepoint the results of the respective treatments were compared to the results from the untreated control using One-way ANOVA followed by Dunnett’s multiple comparison test. *, p < 0.01; **, p < 0.01; ***, p < 0.001; ****, p < 0.0001.

The quantitative results of the neurite network-staining are shown in Figure 6 that presents the average neurite network area per cell normalized to untreated control. Fish Gelatin caused an apparent decrease in the neurite network size per cell at a single concentration (10% w/w) after 24 h of exposure but otherwise caused no significant effects on the amount of the neurite network at any timepoint or concentration, although it did demonstrate an apparent, but insignificant, time- and dose-dependent increase in neurite network area (Figure 6, green bars).

Exposure to AM and A23187 caused a near-complete absence of the neurite network area per cell in a time- and/or dose-dependent manner (Figure 6 brown and red bars), whereas Tunicamycin caused only a minor time- and dose-dependent decrease (Figure 6, blue bars).

The 2.5 iPAAG, at concentrations up to 20% (w/w) for up to 96 h of exposure, did not affect the amount of the neurite network per cell significantly, but like Fish Gelatin it did demonstrate an apparent, but insignificant, time- and dose-dependent increase in neurite network formation.

The data presented in Figure 6 are supported by the β3-Tubulin stains presented in Figure 3 where fasciculated neurites are clearly identifiable in control cultures and cultures exposed to Gelatin, and 2.5 iPAAG. Cultures exposed to Tunicamycin also demonstrate fasciculated neurites, but the network is less elaborate than in control-, Gelatin-, and 2.5 iPAAG-exposed cultures. In cultures exposed to AM and A23187, no fasciculated neurites are identifiable.

In summary, at the tested concentrations and timepoints, Fish Gelatin had no or minor effects on the formation and maintenance of the neurite network, whereas Tunicamycin caused a minor time- and dose-dependent decrease in neurite network formation, but did not abrogate the process. In contrast, AM and A23187 caused pronounced time- and dose-dependent decreases in neurite network formation, leading to an abrogation of the process.

The 2.5 iPAAG, at concentrations up to 20% (w/w) for up to 96 h of exposure, had no statistically significant effects on the neurite network area, although it, like Fish Gelatin, demonstrated a tendency towards a time- and dose-dependent increase in neurite network formation.

## 4 Discussion

Arthrosamid^®^ and Bulkamid^®^ are established human medical devices marketed for several years with a documented high efficacy and low degree of adverse events (Henriksen et al., 2023; Hoe et al., 2021; Tnibar, 2022).

The 2.5 iPAAG products have passed biocompatibility testing including testing of cytotoxicity, sensitization, irritation or intracutaneous reactivity, material mediated pyrogenicity, acute systemic toxicity, sub-acute toxicity, sub-chronic toxicity, chronic toxicity, genotoxicity, carcinogenicity, reproductive and developmental toxicity, degradability testing, as well as leachables and extractables testing. Nevertheless, some regulatory authorities have occasionally argued that the pain-reducing effects of products like Arthrosamid^®^ and Arthramid^®^ could be due to a neurotoxic effect of the products.

During production of the 2.5 iPAAG, unpolymerized AM and MBAM remaining in the gel after the polymerization process are removed by a lengthy washing procedure, reducing the residual concentrations of AM and MBAM in the final product to ≤1.5 mg/kg and ≤1 mg/kg, respectively. For a standard 6-mL treatment of a human knee with Arthrosamid^®^ this corresponds to a maximum administration of 9 µg AM and 6 μg MBAM, respectively. For comparison, the estimated mean dietary exposure to AM ranged from 0.2 to 1.0 μg/kg bw per day for the general adult population (Joint_FAO/WHO_Expert_Committee_on_Food_Additives, 2011).

In addition to the AM remaining in the hydrogel after the manufacturing process, additional AM can theoretically be generated as a by-product of polyacrylamide degradation mediated by thermal, photo-, biological, chemical, and mechanical means. The general process of polyacrylamide degradation has been investigated in detail by, e.g., Caulfield et al. (Caulfield et al., 2003a; Caulfield et al. 2003b; Caulfield et al., 2002) who conclude that polyacrylamides are nontoxic and do not undergo unzipping type reactions to form significant amounts of AM. Notably, many of the conditions facilitating degradation (high temperatures, acidic or alkaline conditions, UV exposure) are not physiologically relevant, and any degradation of 2.5 iPAAG following soft tissue or intra-articular implantation is therefore expected to be negligible. This is supported by studies demonstrating, e.g., that intra-articularly injected 2.5 iPAAG is incorporated into the synovial membrane which maintains its increased volume for at least 2 years following injection (Christensen et al., 2016) and studies demonstrating that treatment of urinary incontinence by bulking with 2.5 iPAAG can remain effective for at least 7 years (Brosche et al., 2021).

To clarify whether the 2.5 iPAAG has any neurotoxic effects *in vitro*, neuronal cultures of iCell^®^ GlutaNeurons were exposed to 2.5 iPAAG for up to 96 h, using Fish Gelatin, AM, Tunicamycin, and A23187 as controls. Tunicamycin is a well-known neurotoxin (Ko et al., 2020). It inhibits N-linked glycosylation through inhibition of UDP-N-acetylglucosamine-dolichol phosphate N-acetylglucosamine-1-phosphate transferase (GPT) leading to Endoplasmic reticulum stress and subsequent apoptosis (Oslowski and Fumihiko, 2011; Guha et al., 2017). A23187 is an antibiotic and a divalent cation ionophore that induces Ca^2+^-dependent necrosis and apoptosis in a cell-type specific manner (Matsubara et al., 1994; Patel et al., 2023).

Cocultures of iCell^®^ GlutaNeurons with astrocytes can generate functional neuronal networks with spontaneous burst activity (Overland et al., 2017; Tukker et al., 2018; Tukker AnkeM. et al., 2020). However, studies of neuronal excitability were outside the scope of the current study, and therefore a simpler monoculture setup was selected. Such monocultures have previously been used for neurotoxicity studies of, *e.g.*, neurite outgrowth and cell viability (Overland et al., 2017; Cohen and Tanaka, 2018).

Here, cells were plated on a coat of PLO and Matrigel as recommended by the supplier (Cellular_Dynamics_International, 2017). This setup led to cultures consisting of cellular aggregates interconnected with fasciculated neurites (Figure 1). This morphology is comparable to that presented by Overland et al. (2017) (Overland et al., 2017).

In addition to the untreated control cells (cells exposed to the BrainPhys™ Neuronal Medium alone), cells were as a negative control treated with Fish Gelatin in concentrations identical to those used for 2.5 iPAAG. The Fish Gelatin was selected because it has a viscosity comparable to that of the 2.5 iPAAG. However, in contrast to 2.5 iPAAG, which is considered chemically inert, gelatine can be defined as a partially hydrolyzed type of collagen (Schrieber and Gareis, 2007) and may therefore for instance retain binding motifs for integrins, thus facilitating cell-substrate attachment (Davidenko et al., 2016). Consequently, it is not unexpected that Fish Gelatin did demonstrate a tendency towards a time- and dose-dependent stimulation of neurite network formation, since the integrin-binding RGD-motif present in gelatine is known to be able to stimulate neurite outgrowth (Madl and Heilshorn, 2015).

However, like Fish Gelatin, the 2.5 iPAAG also demonstrated a tendency towards a time- and dose-dependent stimulation of neurite network formation suggesting that the effect could be related to the mechanical properties of the gels. Indeed, the rheological properties of substrates are reported to have significant effects on cell behavior, and several studies demonstrate that although polyacrylamide is considered a chemically inert molecule, it can still affect biological processes through mechanical means (Lo et al., 2000; Munakata et al., 2017). For instance, neurons grown on different polyacrylamide hydrogels demonstrate differences in neurite branching with neurons growing on softer substrates forming more branches than those grown on stiffer gels or hard substrates like glass (Flanagan et al., 2002). Therefore, the 2.5 iPAAG can in principle affect neurite network areas formation in iCell^®^ Glutaneuron cell cultures through effects on the neurite outgrowth process.

As expected, Tunicamycin caused a time- and dose-dependent increase in cell death and apoptosis, but surprisingly only a minor decrease in the average neurite network area. Tunicamycin also caused mainly insignificant effects on the number of cells in the cultures, as determined from Hoechst 33342 staining. However, considering the detected increases in the number of dead cells and cells undergoing apoptosis, this may be an artefact caused by the automated detection of Hoechst 33342-stained nuclei in combination with apoptosis-mediated nuclear fragmentation.

The calcium ionophore A23187 is reported to induce time- and dose-dependent cell death in various cell types, caused by both apoptosis and/or necrosis (Matsubara et al., 1994; Petersén et al., 2000), and the compound is also reported to reduce neurite outgrowth in a dose-dependent manner in, *e.g.*, cultured embryonic mouse spinal cord neurons (Bird and Owen, 2000). In cultures of embryonic striatal neurons A23187 is reported to induce cell death through induction of oxidative stress, opening of the mitochondrial permeability transition pore, and activation of caspases (Petersén et al., 2000). Exposure of neurons to A23187 has also been demonstrated to lead to a degeneration of neurites caused by disintegration of their axonal microtubules and neurofilaments (Schlaepfer, 1977).

AM is known to be cytotoxic and neurotoxic as well as being carcinogenic and affecting reproductive and developmental toxicity (Zhao et al., 2022). AM induces both necrotic cell death and apoptosis through various signaling pathways (Liu et al., 2015; Park et al., 2010; Zhao et al., 2022) and neurotoxicity through oxidative stress (Kopańska et al., 2022; Zhao et al., 2022), and even at non-cytotoxic concentrations AM is reported to reduce the number of neurites per cell (Nordin-Andersson et al., 2003). The neurotoxic effects of AM seems to be caused by specific effects on nerve endings disrupting presynaptic nitric oxide (NO) signaling and neurotransmitter release (LoPachin and Terrence, 2008), affecting cytoskeletal proteins, and eventually causing neurite degeneration (Spencer et Spencer and Schaumburg, 1975; Zhao et al., 2022).

In the present study, AM caused a pronounced time- and dose-dependent decrease in the number of cultures cells, an increase in cell death, but not in Caspase-3-mediated apoptosis. Moreover, the compound caused a strong decrease in neurite network area formation. The effects largely mimic those observed after exposure of the iCell^®^ Glutaneurons to medium-to-high concentrations of A23187, and AM and A23187 do seem to affect some of the same cellular processes causing, *e.g.*, oxidative stress and effects on cytoskeletal elements. In contrast, the neurotoxic effects of Tunicamycin seem to be caused through different pathways.

Importantly, the effects of exposure of iCell^®^ Glutaneurons to different concentrations of AM, A23187, and Tunicamycin do not resemble the effects observed by exposing the cells to 2.5 iPAAG. The hydrogel did not at any tested concentration or timepoint affect any of the estimated parameters in a way significantly different from that of untreated control.

In conclusion, the negative control, Fish Gelatin, did not affect the detected number of cultured cells during the 96-h exposure time, and consistently, the compound had no effects on the detected cell death or degree of Caspase-3-mediated apoptosis. The compound had no or minor effects on the formation and maintenance of the neurite network.

The positive controls AM, A23187, and Tunicamycin all demonstrated the expected cytotoxic effects, but also revealed differences in their mode of action. AM and A23187 both caused cytotoxic effects identifiable as a pronounced time- and dose-dependent decrease in the number of cultured cells accompanied by a time- and dose-dependent increase in the amount of detected dead cells and a decrease in the average neurite network area. However, the compounds had no significant effects on Caspase-3-mediated apoptosis. In contrast, Tunicamycin caused a time- and dose-dependent increase in the number of detected dead cells accompanied by a time- and dose-dependent increase in Caspase-3-mediated apoptosis and a minor decrease in the average neurite network area.

At the tested concentrations and timepoints, up to 20% (w/w) for up to 96 h, the 2.5 iPAAG had no statistically significant effects on the number of cultured cells, the amount of detected dead cells, the degree of Caspase-3-mediated apoptosis, or the amount of neurite network area per cells. However, the compound did demonstrate a tendency towards a time- and dose-dependent effect on the neurite network formation. These results document that the 2.5 iPAAG included in medical devices and veterinary medicinal products manufactured by Contura demonstrate no apparent cytotoxic or neurotoxic effects in human iCell^®^ Glutaneurons at the tested concentrations for up to 96 h of growth *in vitro*.

## Data Availability

The raw data supporting the conclusions of this article will be made available by the authors, without undue reservation.
